# A cross-cultural study to identify social behaviours of pedestrians in urban public spaces: evidence from Iran, Spain, Italy, and Australia

**DOI:** 10.1038/s41598-025-16421-7

**Published:** 2025-08-26

**Authors:** Reza Askarizad, Patxi J. Lamíquiz-Daudén, Mana Dastoum, Elham Mehrinejad Khotbehsara, Ayyoob Sharifi, Chiara Garau

**Affiliations:** 1 Department of Civil and Environmental Engineering and Architecture (DICAAR), University of Cagliari, Via Marengo 2, Cagliari, 09123 Italy; 2 Department of Urban and Regional Planning, Universidad Politécnica de Madrid, Madrid, 28040 Spain; 3 Department of Construction and Architectural Technology, School of Architecture (ETSAM), Universidad Politécnica de Madrid (UPM), Madrid, Spain; 4 Faculty of Health, Engineering and Sciences, University of Southern Queensland, Toowoomba, Australia; 5 The IDEC Institute & Network for Education and Research on Peace and Sustainability (NERPS), Hiroshima University, Higashi-Hiroshima, Japan; 6 School of Architecture, Design and Planning, The University of Queensland, Brisbane, 4067 Australia

**Keywords:** Cross-cultural study, Pedestrian social behaviours, Social interactions, Space syntax, Urban public squares, Psychology and behaviour, Sustainability

## Abstract

Despite the growing emphasis on social sustainability in urban design, limited research has examined how spatial configurations influence socio-behavioural patterns across culturally distinct urban squares. This study addresses this gap by investigating how the spatial configuration of public squares interacts with pedestrian social behaviours in four cities in Iran, Spain, Italy, and Australia. Guided by theories of space syntax and social behaviour frameworks, a unique mixed-method approach was adopted, combining spatial configuration analysis, behavioural mapping, and people-tracing, coupled with agent-based simulations. The findings revealed that the accessibility of urban squares is not necessarily determinative of their social vibrancy. Rather, stimulating sustained social life in these environments is governed by an intricate nexus of factors, including culturally specific behaviours, the purposeful allocation of functional amenities, and the critical amelioration of socio-economic challenges inherent to their shared public domain. Notably, this cross-cultural analysis highlights how socio-spatial dynamics differ across contexts, offering a richer lens for inclusive design. The originality of this study lies in its multi-scalar, comparative, and culture-sensitive approach to analysing public squares, advancing urban design strategies that are adaptable, equitable, and responsive to both spatial logic and cultural diversity.

## Introduction

Social behaviours in urban spaces are an indispensable part of public life, reflecting how individuals and groups interact with one another and with their surrounding environment. These behaviours not only shape the vibrancy of urban spaces but also contribute to their social, cultural, and functional significance^1^. Understanding the behavioural patterns of these interactions is crucial for urban planning and design, as it provides insights into the patterns of use, the formation of social bonds, and the collective identity of communities^2^. However, the way people behave in urban spaces is not universal; it is profoundly influenced by cultural contexts, which dictate norms, values, and spatial preferences^3,4^. For example, an urban public square in a country like Iran may prioritise privacy, personal space, gender separation, and solitary activities^5–7^ while one in others like Italy or Spain may emphasise openness, communal interaction, and shared experiences, which may manifest in more frequent social gatherings and group-based behaviours in public spaces^8,9^. Exploring these variations is essential to unveiling how different societies adapt to the unique needs and values of diverse communities^10,11^. Such knowledge offers invaluable guidance for creating spaces that support diverse cultural expressions and cultivate public engagement^12^.

The spatial configuration of urban public spaces plays a crucial role in shaping and guiding social behaviours, as it determines how individuals and groups navigate, perceive, and utilise these spaces^13,14^. Through the arrangement of streets, alleys, squares, and behavioural settings, urban design can facilitate or impede social interactions, influence movement patterns, and define areas for gathering or solitude^15^. Space syntax, a well-regarded analytical tool in urban studies, provides a framework for understanding the relationship between spatial layout and human behaviour, by formulating the implicit cognitive maps that pedestrians use to navigate urban environments^16^.

By examining topological relations, including connectivity, integrity, and visibility, within the built environment, it becomes clear how spatial configurations influence socio-cultural and behavioural patterns across different contexts^17,18^. This approach raises a couple of crucial questions: Are there significant differences in the established socio-behavioural patterns of pedestrians in public urban squares? Does spatial configuration primarily shape socio-behavioural patterns, or do cultural particularities exert a greater influence? Addressing these questions requires a nuanced understanding of the interaction between spatial design and behavioural patterns in different contexts, offering a pathway to designing urban spaces that are not only functional but also culturally resonant.

To address these crucial questions, particularly regarding the variations in pedestrian socio-behavioural patterns, this study focuses on urban public squares. Urban public squares serve as vital social arenas where people gather, interact, and engage in a diverse range of activities^19,20^ reflecting the evolving interplay between spatial configuration and socio-behavioural interactions. The social behaviours established in these spaces not only shape the character of public life but also reveal deeper insights into the unique cultural norms, values, and socio-spatial practices of each specific context. Recognising these behavioural differences is indispensable for designing responsive urban environments that accommodate the distinct needs of their communities.

While urban studies have extensively explored pedestrian behaviours in sociable public spaces, most of these investigations are restricted to single-case or single-context analyses, often lacking a comparative lens across different cultural and morphological settings^21–24^. Few studies have systematically examined how spatial configuration interacts with culturally ingrained behavioural patterns, leaving a gap in understanding how universal design principles can be adapted to distinct socio-cultural contexts. Moreover, cross-cultural analyses that integrate both quantitative spatial metrics, such as space syntax, and qualitative behavioural observations are notably scarce, despite their potential to offer a holistic and grounded understanding of urban social life. This study bridges this critical gap by examining pedestrian behaviours in urban squares across four culturally diverse contexts, including Iran, Spain, Italy, and Australia incorporating spatial analysis and empirical observations to uncover cultural particularities and shared patterns in socio-behavioural practices.

This study aims to investigate the socio-behavioural patterns of pedestrians in urban public spaces across four distinct cultural contexts—Iran, Spain, Italy, and Australia—by examining the interaction between spatial configuration and socio-behavioural interactions. Through a combination of space syntax analysis and empirical observations, the research aims to identify both universal and context-specific behavioural traits, offering insights into how urban design can accommodate diverse cultural needs while fostering purposeful urban vitality.

The research outline in this study will be followed by an explanation of the methodology employed, including conceptual framework, space syntax analysis, systematic observational techniques, and the selection of case studies from Iran, Spain, Italy, and Australia. The findings from these analyses will be presented, comparing the context-specific socio-behavioural patterns across the spatial configuration of the four different contexts. The Discussion Section will interpret these results in light of the hypothesis regarding the relative influence of spatial configuration versus cultural variations. Finally, the paper will conclude by summarising key insights and offering recommendations for culturally sensitive urban design, as well as future cross-cultural research in public spaces.

## Materials and methods

This study adopts a mixed-method approach, combining quantitative spatial analysis and qualitative empirical observation to explore the socio-behavioural patterns of pedestrians in urban public spaces. To ensure a structured and rigorous analysis, a conceptual framework was initially developed based on the study’s aims, objectives, and key variables deemed influential to legitimise and support the adopted procedures throughout the analysis process. Accordingly, the methodological approach integrates spatial configuration analysis through space syntax and systematic behavioural observation techniques. Following the analysis, the consistency between the configurational attributes of the public squares and the associated behavioural patterns was examined. Finally, a comparative analysis was performed to identify socio-behavioural variations in pedestrian activities within urban public spaces across the selected cultural contexts. The subsequent sections provide a detailed description of these underpinning procedures.

### Conceptual framework

This conceptual framework explores the drivers of social behaviour in urban public spaces, highlighting the reciprocity between spatial configuration, visibility, and social activities as key determinants of pedestrian social engagement. Spatial configuration significantly influences how people perceive, navigate, and use urban spaces^17^. Rooted in the principles of space syntax^13^ this theoretical foundation postulates that the spatial layout of urban spaces shapes movement patterns, social encounters, and behavioural tendencies. Hillier^25^ articulated that the distinctive character of a settlement’s built environment arises from its socio-cultural context, which imposes specific spatial constraints. Building on this concept, space syntax provides a quantitative framework for analysing the connectivity, integration, and visibility of urban layouts, enabling a deeper understanding of the cognitive maps pedestrians use when navigating urban public spaces^16^. Central to this approach is the hypothesis that spatial configurations act as both enablers and constraints for social encounters. Highly integrated spaces attract more intense pedestrian flow and encourage spontaneous social interactions, while segregated spaces may limit accessibility and reduce opportunities for collective engagement^15^.

Urban design theorists further argue that the arrangement of spatial configuration shapes individuals’ cognitive maps, and behavioural tendencies, enabling the formation of clear mental images that determine how people navigate and interact within legible urban spaces^26–29^. In urban public spaces, spatial configuration not only orients movement dynamics but also defines gathering areas and the overall sociability of the space^30^. Additionally, social behaviours are strongly influenced by visibility within a given environment^31^. Essentially, who sees whom, and who is seen by whom, shapes how people interact. A vibrant urban space is often contingent upon the ability of people to see, hear, and interact with one another^32^. The attractiveness of such spaces hinges on these possibilities for social engagement. This spatial analysis establishes the basis for understanding how configurational attributes interact with socio-cultural patterns to produce diverse socio-behavioural outcomes^33^.

Visibility Graph Analysis (VGA) within the framework of space syntax offers a substantial instrument for understanding the role of visibility in shaping social behaviours within the built environment. VGA measures the visual permeability between different points in a spatial configuration, allowing researchers to map out areas of visual accessibility and detect visual obstacles that may limit social engagement^34^. These obstacles can be categorised into two main types: accessibility obstacles, which physically restrict pedestrian movement, and visibility obstacles, which block the visual field without necessarily impeding physical access. Visibility obstacles include elements such as urban furniture, landscaping features, statues, kiosks, or any artifact positioned at human eye level that interrupts sightlines within the public space. By simulating how an individual perceives their environment at a standing height, VGA provides a perspective on how open or fragmented a space appears to its users. According to Hillier^14^, Turner^35^ and previously elaborated by Gehl^32^ spaces with greater visual permeability tend to facilitate higher levels of natural movement and social interaction, as users are more likely to move toward, pause, and engage in areas that are visually rich and easily understandable. Therefore, VGA not only reveals the structural affordances of the built environment but also reveals the socio-spatial mechanisms through which visibility promotes opportunities for social encounters and communal engagement.

Gehl^32^ categorised outdoor activities into three types: necessary, optional, and social, with the latter considered the most important one. Social activities, as defined by Unger and Wandersman^36^ and further elaborated by Huang^37^ involve interactions ranging from active conversations to passive observations. These social interactions are crucial for the socialisation process within urban spaces, as emphasised by Forgas^38^. Spaces that facilitate static activities such as sitting, eating, and reading encourage casual and low-pressure social behaviours, which can provide opportunities for brief encounters and relaxed exchanges with others^39,40^. Numerous studies have validated that engaging in social interactions and establishing positive social behaviours can lead to overall physical and mental well-being^41–43^.

Urban public squares are central to the social, cultural, and functional heartbeat of cities, serving as hubs for interaction, identity formation, and diverse activities^44^. Lang and Marshall^20^ highlight the importance of public squares as effective behaviour settings, emphasising that their design must facilitate a variety of interactions and activities. Squares function as spaces where individuals and groups engage in specific behaviours, whether informal (e.g., leisure, socialising) or formal (e.g., events, markets)^45^. To support these behaviours, squares must provide accessibility, activity diversity, and amenities^20^. Accessibility entails physical and visual connectivity to the square, ensuring usability and integration with the urban fabric. Squares offering multiple behaviour settings—such as areas for sitting, walking, interacting, eating, or performing—encourage greater social engagement, cultivating activity diversity. Additionally, physical attributes and amenities, such as seating arrangements, shaded areas, and open spaces, must align with user needs and cultural expectations to enhance usability. According to Lang and Marshall^20^ successful squares support a critical mass of users by accommodating diverse behaviours and promoting sociability.

**Fig. 1:**
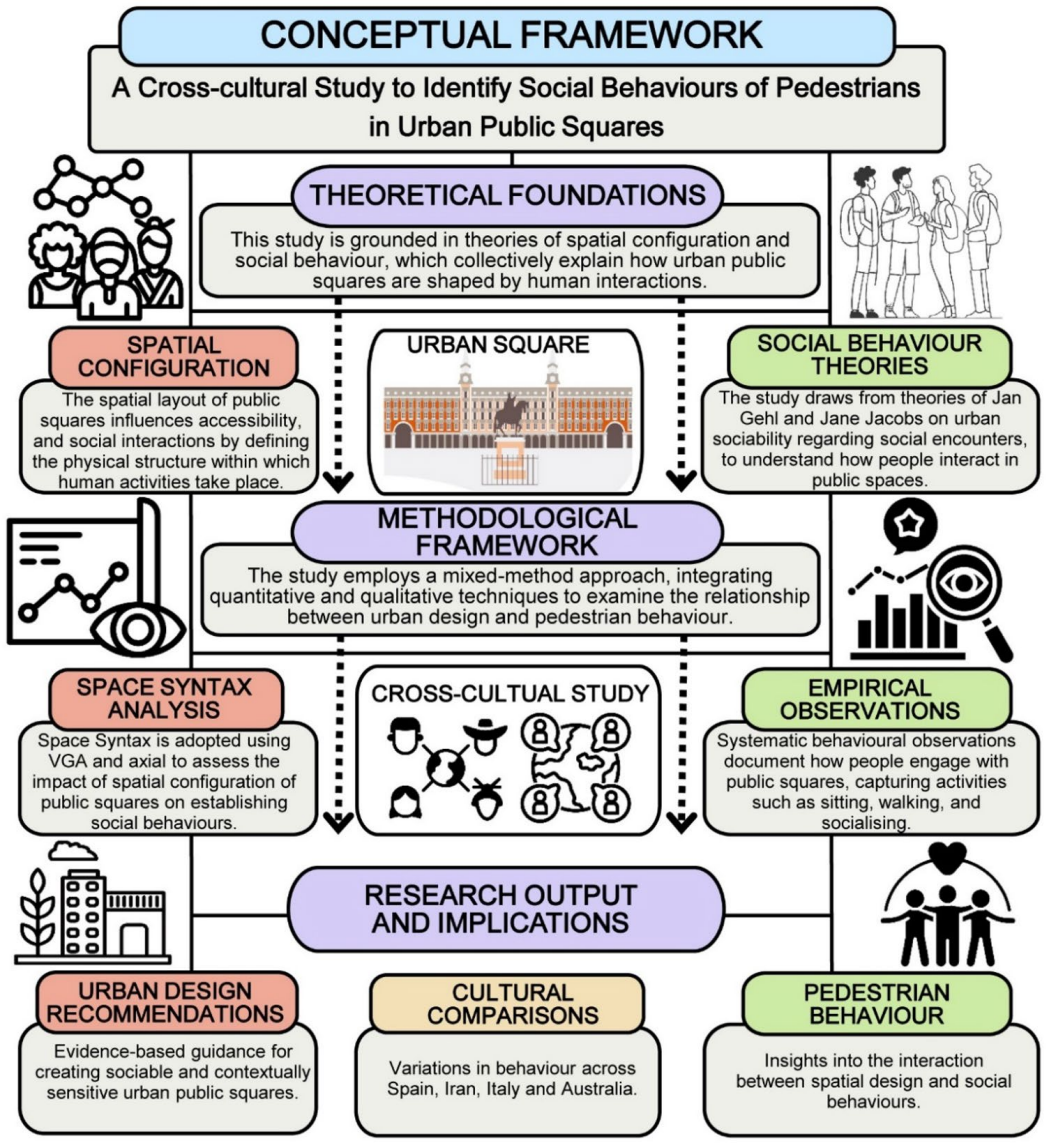
Conceptual framework developed in this study, illustrating the relationship between the theoretical foundation, methodological framework, and the expected research output (Developed by corresponding author and P.J.L.D.).

In sum, urban squares act as vital arenas where design and socio-behavioural activities intersect to shape human interactions. To address the complexities of socio-behavioural patterns in urban public squares across various case studies, this study incorporates a multidimensional conceptual framework. Drawing on the aforementioned theoretical underpinnings, this research employs space syntax analysis and systematic behavioural observations to unveil the interaction between spatial design and social behaviours in various cultural contexts. Figure 1 illustrates the conceptual framework developed, focusing on the relationship between configurational attributes and pedestrian behaviours within urban public spaces.

### Spatial configuration analysis using space syntax

The first methodological layer involves the application of space syntax to analyse the spatial configurations of urban public squares at two distinct levels. First, at the neighbourhood scale, the analysis considers the public squares within their broader urban context, including the surrounding street network and buildings. Axial line analysis is employed here to examine key variables that influence pedestrian accessibility and movement patterns. Space syntax has been widely recognised for its ability to quantify spatial properties, particularly in relation to pedestrian flows and socio-spatial interactions^13,15^. This analysis provides an understanding of how the surrounding urban fabric impacts pedestrian flow to and from public squares. Second, at the square scale, the focus shifts to the internal spatial properties of the individual public square itself. VGA and agent-based simulations assess elements including visibility, spatial hierarchy, and interaction opportunities, which are shaped by visual fields. These approaches have been successfully applied in studies linking visibility to movement and social behaviour^34,46^. The inclusion of spatial metrics such as the level of depth, visual connectivity, control, and clustering highlights the role of design elements in fostering or hindering social behaviours.

The space syntax analysis in this study was performed using Depthmap 10 software, following a set of sequential procedures. First, the urban plans of the study areas were drawn in AutoCAD 2010, centring on the targeted public squares. These drawings were completed within the neighbourhood-scale proximity to cover potential pedestrian trajectories leading to the public plazas. Next, the two-dimensional plans were converted into DXF format to meet the software’s requirements for generating syntactical graphs. Once imported into Depthmap 10, the analytical process commenced. For axial line map analysis at the neighbourhood scale, variables such as integration, connectivity, choice, and control were assessed to provide an overview of the role of the spatial structure in influencing pedestrian movement.

Spaces that are more integrated within a network tend to be used more often^47^ and greater connectivity signifies easier access^48,49^. The choice variable illustrates trajectories with shorter paths to the destination, while control assesses the degree to which a space controls access to its immediate neighbours^47^. Locations where the view changes significantly are likely to become resting spots, as indicated by a high visual clustering coefficient^34,50^. Visual control quantifies the potential of a space to observe and be observed, where higher control values may imply enhanced informal surveillance^35,51^. In contrast, agent-based analysis focuses on simulating the movement behaviour of virtual agents within a 3D spatial environment defined by VGA parameters. Agent-based modelling has been widely applied in urban studies to explore potential pedestrian dynamics and accessibility patterns^52,53^. This allows researchers to analyse how spatial configurations influence pedestrian flow and socio-behavioural patterns^47,54^. According to Gehl’s framework of outdoor activities^32,55^ enabling such visual attributes has significant implications for social interaction, privacy, and security, potentially leading to the identification of hotspots for optimised surveillance.

In the next step, the detailed layout of the targeted urban squares was redrawn in AutoCAD, with particular attention to urban design elements such as urban furniture and both natural and artifacts that could potentially interrupt movement patterns or influence social encounters. Such a modelling process comprised two disparate categorisations, entailing both accessibility barriers and visual barriers. Accessibility obstacles focus on physically restricting pedestrian movement, while visibility obstacles focus on blocking the visual field without necessarily impeding physical access. By comparing the differences between how an individual perceives their environment at a standing height and their available trajectories to move toward their destination, VGA provides a perspective on how the built environment appears to its users. Afterward, multiple agents were animated from the entry points to discern the relationship between their visual field and their intended directions of movement. These analytical steps were applied to each case study to systematically investigate the influence of spatial layout on the establishment of social behaviours in various cultural contexts.

### Empirical observations of social behaviours

The second phase of analysis comprises systematic empirical observations aimed at validating and complementing the findings from the spatial configuration analysis. The primary objective is to establish a relationship between the syntactical attributes of public squares and the behavioural patterns observed within them. Furthermore, this phase seeks to document and analyse the diversity of social behaviours established by pedestrians in these spaces. Behavioural mapping and people-tracing methods have been widely adopted to study the influence of spatial design on pedestrian behaviour^2,47,55,56^. Observations were conducted using the static snapshot, dynamic video recording, as well as people-tracing methods, supplemented with behavioural mapping techniques to capture the range of pedestrian activities occurring in the squares.

A purposive sampling strategy was applied for people-tracing observations to ensure diversity across gender and age groups, with an equal representation of eight male and eight female participants per site. This purposive approach aligned with the conceptual framework of the study, which sought to explore behavioural differences across demographic categories and identify culturally specific patterns of pedestrian activity.

In documenting behavioural maps, specific geometric shapes are used to visualise each behavioural pattern, allowing for easy distinction based on the frequency of activities observed within the targeted settings. Behaviours such as sitting, standing, engaging in social interactions, eating and drinking, playing, smoking, taking photos or videos, and using mobile devices were systematically recorded. These observations were then translated into detailed behavioural maps to identify patterns of activity distribution and social engagement established by pedestrians. To ensure comparability across case studies, each site was observed during weekdays under similar weather conditions and at consistent time frames within the evening, minimising the influence of intervening variables such as extreme weather or weekend events. However, a single time frame was selected for each square due to logistical constraints.

According to Gehl’s theory of outdoor activities^32,45^pedestrian movement in urban spaces is categorised into three descriptive types relevant to this study: necessary activities, optional activities, and social activities. Necessary activities include obligatory journeys like commuting or essential errands, occurring regardless of the environment’s quality. Optional activities describe chosen behaviours such as leisurely walks, outdoor relaxing or passive recreation, highly influenced by the attractiveness and comfort of the urban space. Finally, social activities refer to actions arising from the presence and interaction of people in public areas, facilitated by well-designed spaces that encourage congregation and interaction. This framework facilitates the analysis of how urban design impacts different forms of pedestrian activity, suggesting the distinction between essential travel and the more discretionary uses of public space using people-tracing method. In addition to this behavioural categorisation, the observation process further classified pedestrian activities through their demographic data such as gender and age. This classification approach is consistent with other empirical studies of urban behaviour^2,56^. This multi-layered classification system, grounded in Gehl’s foundational work, provides a comprehensive framework for analysing pedestrian behaviour within the studied urban spaces.

Through overlaying the results of these methodological layers, space syntax, simulation analysis, and empirical observations, the study aims to identify correlations between spatial configurations and observed behavioural patterns within the different studied urban squares. This integration enables a detailed interpretation of how spatial design and social activities interact to shape pedestrian behaviour, ensuring a balanced exploration of social, spatial, and behavioural analysis. Furthermore, by incorporating spatial analysis, and empirical observations, this study contributes to the broader discourse on cross-cultural urbanism and inclusive urban design practices. Ultimately, this established methodological framework serves to deepen our understanding of how public urban squares function as social spaces in culturally distinct contexts.

### Study areas and case studies

The research focuses on four cities: Isfahan in Iran, Madrid in Spain, Cagliari in Italy, and Brisbane in Australia. The selection of these countries was guided by their contrasting cultural backgrounds and rich urban and architectural histories, which provide a diverse range of socio-spatial patterns. Within these cities, the study selects the prominent public squares that are both architecturally significant and serve as critical hubs of social activity. Furthermore, the study specifically selected squares characterising varied and diverse scale and spatial configuration typologies, with the explicit aim of enhancing the broader applicability of the findings. The chosen case studies, namely Naghshe Jahan Square in Isfahan, Plaza Mayor in Madrid, Bastione di Saint Remy in Cagliari, and King George Square in Brisbane, are culturally and functionally important, attracting diverse user groups and hosting a variety of social behaviours. It is worth noting that all the selected case studies are not only among the most significant landmarks of their respective cities but also among the most popular tourist destinations in these locations. Their distinct spatial layouts and cultural contexts make them ideal sites for cross-cultural analysis. In the following subsections, each of these case studies is elaborated in detail, highlighting their inherent particularities.

### Naghshe Jahan Square, Isfahan, Iran

Naghshe Jahan Square, located in Isfahan, Iran, is one of the largest and most historically significant public squares in the world, designated as a UNESCO World Heritage Site (Fig. 3). Built during the Safavid dynasty (16th −17th century), it serves as a multifunctional urban space, integrating religious, commercial, and social activities. The square is surrounded by monumental structures, including the Shah Mosque, Sheikh Lotfollah Mosque, Ali Qapu Palace, and the Grand Bazaar, reflecting the cultural and architectural heritage of Persian urbanism. It functions as a central gathering space for both locals and tourists, facilitating social interactions, festivals, and traditional performances. Its spatial configuration, with a vast open central area enclosed by arcades and monumental buildings, creates a unique pedestrian vibrancy, promoting both movement and socialisation. The square’s integration with the surrounding bazaar and historical landmarks enhances its charm for sociability and commercial liveliness, making it an ideal place for examining how spatial design influences pedestrian behaviour within a culturally and historically significant setting.

### Plaza Mayor, Madrid, Spain

Plaza Mayor, located in the heart of Madrid, Spain, is one of the most historically and culturally significant public squares in the city. Originally built in the 16th century during the Habsburg period, it has served as a central hub for markets, public gatherings, royal ceremonies, and cultural festivities for centuries. Defined by its symmetrical enclosed layout, the square is surrounded by uniform three-story buildings with arcaded walkways, creating a distinct sense of enclosure and spatial order. The large open space at its centre serves as a focal point for pedestrian movement, encouraging both social interaction and temporary event-based activities such as concerts, festivals, Christmas markets, and public celebrations. Its historical monuments, including the statue of King Philip III, add to its symbolic and cultural significance. Plaza Mayor remains a major tourist attraction and a vital part of Madrid’s urban identity, suggesting a unique setting for studying the relationship between spatial configuration, pedestrian behaviour, and cultural dynamics in a historically layered public urban square.

### Bastione di Saint Remy, Cagliari, Italy

Bastione di Saint Remy, situated in the historic district of Castello in Cagliari, Italy, is one of the city’s most prominent landmarks, offering panoramic views of the urban landscape. Originally built as a defensive structure in the late 19th century, it has since been transformed into a multifunctional public space, attracting both locals and tourists for leisure, social gatherings, and cultural events. Its elevated position and architectural composition, featuring grand staircases and terraces, define its spatial character, allowing for varied pedestrian flows and social gathering spots. The square plays a key role in local cultural identity, serving as a venue for open-air exhibitions, performances, and social interactions. Unlike conventional urban squares with direct street-level accessibility, Bastione di Saint Remy incorporates vertical spatial dynamics, influencing the way people navigate, interact, and engage with the space, despite having a direct connection to the historic context of the city. The combination of historical significance, strategic visibility, and diverse user engagement makes it an exceptional case study for understanding how spatial hierarchy and topography affect pedestrian behaviour and social activities in public squares.

### King George Square, Brisbane, Australia

**Fig. 2:**
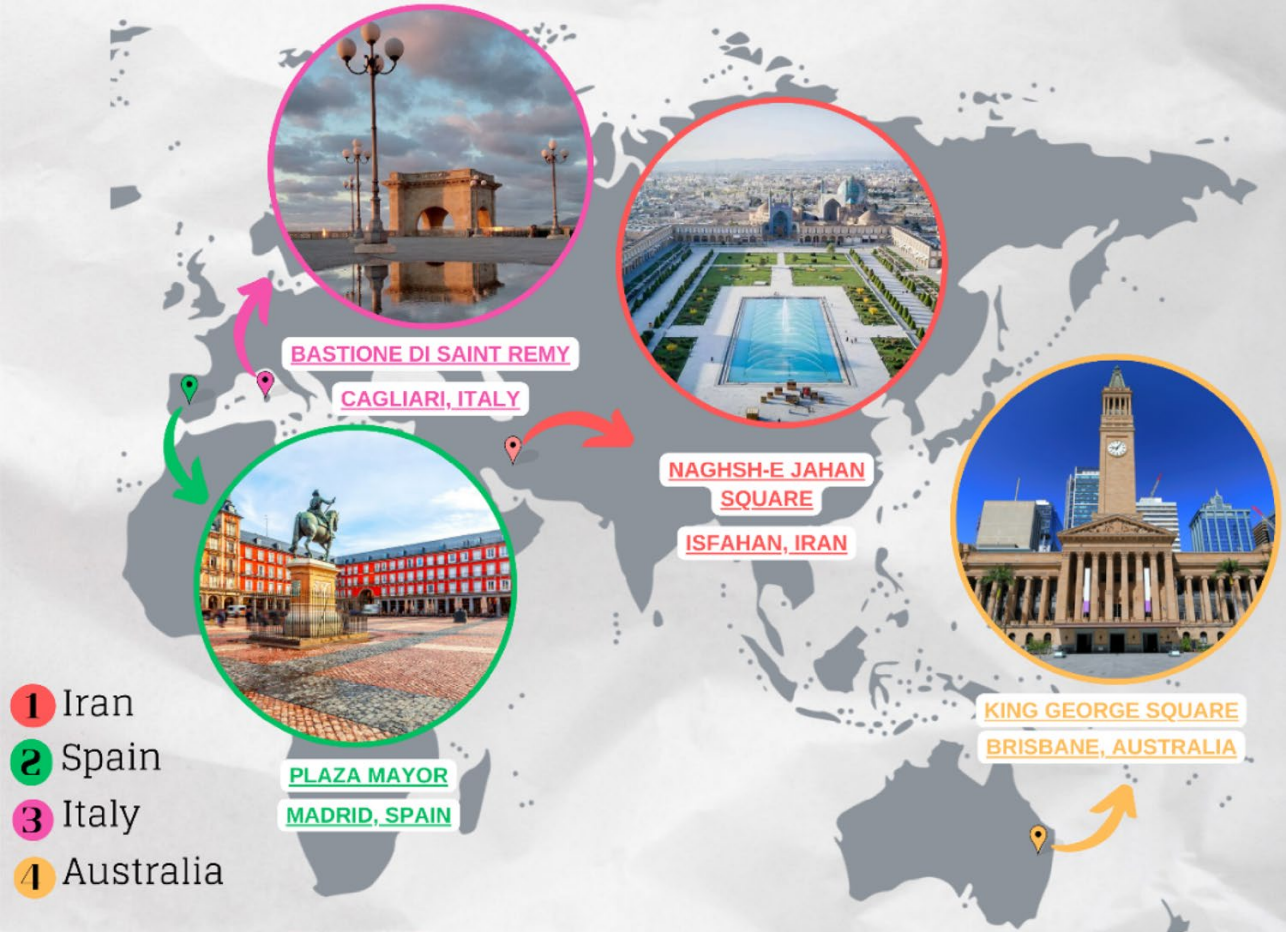
Geographical location of the case studies, highlighting the selected public squares within their respective cities: Naghshe Jahan Square in Isfahan, Plaza Mayor in Madrid, Bastione di Saint Remy in Cagliari, and King George Square in Brisbane (Developed by corresponding author).

King George Square, located in Brisbane, Australia, is one of the largest and most active public squares in the city, positioned adjacent to the Brisbane City Hall. As a central gathering space, it hosts a variety of civic, social, and cultural events, reinforcing its role as an inclusive and adaptable public space. Historically, the square has undergone multiple redevelopments, with the latest transformation aimed at enhancing walkability, accessibility, and social engagement. Unlike traditional European squares defined by architectural enclosure, King George Square features a more open and contemporary design, integrating wide pedestrian walkways, seating areas, and urban greenery to foster interaction and leisure activities. The space is used for markets, concerts, political demonstrations, and festive celebrations, reflecting its multifunctionality. Due to its location in Brisbane’s central business district, the square experiences high pedestrian movement, making it an ideal site for examining urban sociability, spatial accessibility, and behavioural diversity. Its modern urban design, functional adaptability, and strong community presence make it a compelling case study for understanding social behaviours in contemporary public squares. Through the examination of these four public squares, this study entails a diverse range of spatial configurations, social behaviours, and cultural influences, thereby offering valuable implications into how urban design shapes pedestrian interactions and community engagement across different cultural contexts. Figure 2 depicts the geographical location of each selected case study on the map.

### Analysis and results

#### Spatial configuration analysis using space syntax

The analysis begins by identifying the key spatial configuration differences among the study areas to clarify the morphological characteristics of each urban context. In the Iranian case (Isfahan), the surrounding urban texture can be categorised into two distinct patterns: the alleys and peripheral streets, which exhibit an organic configuration, and the main roads and streets, which follow a more grid-like and structured pattern. Consequently, the study area in Isfahan represents a semi-urbanised planning system, blending traditional organic morphology with modern urbanised layouts. Similarly, the historical core of the Spanish case (Madrid) features a semi-organic configuration, reflecting the evolution of its medieval street network while incorporating more structured urban elements. In contrast, the Italian case (Cagliari) is predominantly characterised by an organic urban fabric, with irregular, winding streets and compact spatial arrangements. However, the Australian case (Brisbane) stands out with its highly structured, grid-based configuration, marked by larger urban blocks and wider streets, reflecting modern planning principles. These differentiations in spatial configuration notably influence movement patterns and social behaviours, as further analysed in the subsequent syntactical evaluations. The overall spatial configurations of the study areas are summarised in Table 1.

**Table 1:**
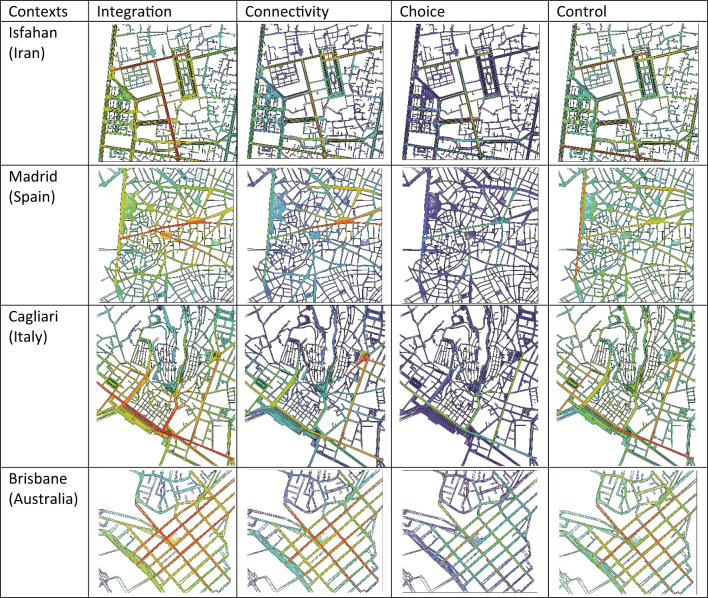
Spatial configuration and obtained syntactical graphs of the study areas in different contexts revealing higher values indicated in red and lower values, indicated in blue (Developed by corresponding author).

**Fig. 3:**
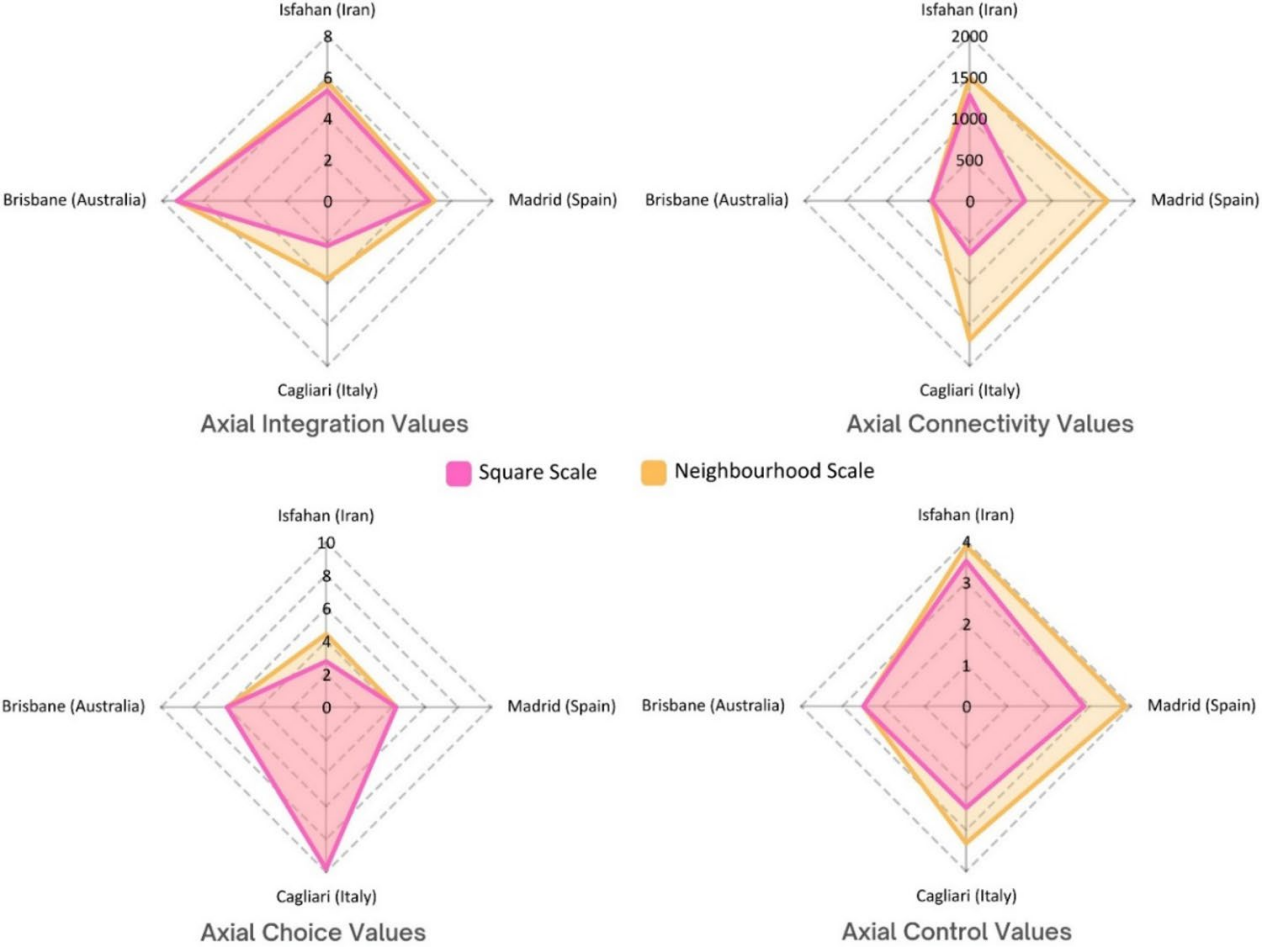
Comparative radar charts illustrating the highest syntactical values (integration, connectivity, choice, and control) within the wider neighbourhood scale versus the corresponding values of the targeted urban public squares in Isfahan (Iran), Madrid (Spain), Cagliari (Italy), and Brisbane (Australia) (Developed by corresponding author).

The quantitative analysis of spatial configuration was conducted using space syntax methodology through axial line map at the neighbourhood scale, covering the urban public squares and their surrounding urban fabric. In this regard, four key syntactical metrics, namely integration, connectivity, choice, and control, were considered influential in these analyses. The primary goal of this analysis was to determine whether the targeted urban squares serve as the primary social hubs within their respective neighbourhoods. To examine this, radar charts were generated, comparing the highest syntactical values of the urban squares against the highest values recorded within their wider neighbourhood contexts. These comparative visualisations are presented in Fig. 3.

The results revealed that the typology of urban squares, specifically whether they are enclosed or open, affects the syntactical values and overall sociability of the urban public square. In the Iranian (Isfahan), Spanish (Madrid), and Italian (Cagliari) cases, where urban squares are architecturally enclosed, these spaces do not demonstrate the highest values of integration, connectivity, or control relative to their wider neighbourhoods. In contrast, in the Australian (Brisbane) case, where the square is open-configured, it holds the highest integration, connectivity, and control values within its neighbourhood context, indicating its central role in movement patterns and social interactions. However, the choice value remains relatively consistent across the Spanish, Italian, and Australian cases, suggesting that regardless of spatial enclosure, the movement trajectories through these squares remain identifiable, particularly for those familiar with their surroundings (Fig. 3). These findings highlight the synergy between spatial configuration and social function, emphasizing how urban morphology shapes patterns of accessibility, movement, and sociability in public squares.

**Fig. 4:**
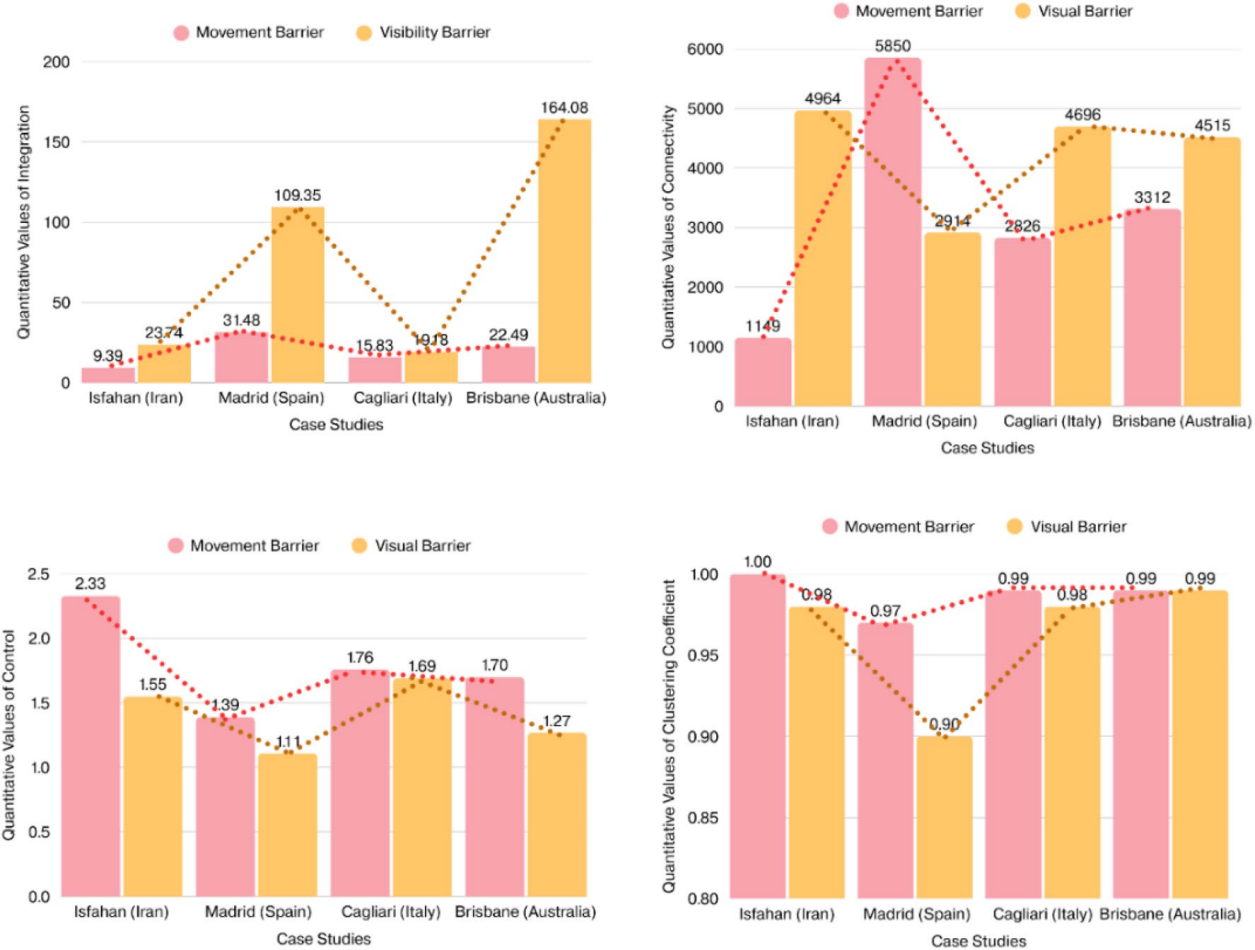
Comparative bar charts illustrating the quantitative values of VGA metrics across the four case studies (Developed by corresponding author).

For the next step of spatial configuration analysis, the detailed analyses across targeted urban squares were conducted through VGA metrics (Fig. 4). Madrid’s Plaza Mayor recorded the highest visual integration (31.48) and connectivity (5850), indicating a highly permeable and integrated visual field. Naghshe Jahan Square in Isfahan showed the lowest visual integration (9.39) and connectivity (1149), suggesting a more segmented and constrained environment. Interestingly, Isfahan led in visual control (2.33), implying stronger potential for passive surveillance, while Madrid recorded the lowest (1.39). Isfahan also had a perfect clustering coefficient (1.00). These results suggest that despite lower integration and connectivity, Isfahan’s internal visual structure may promote focused interactions in more controlled settings, with its Persian garden design prototype providing higher levels of private segments for social gathering. Overall, VGA metrics reveal how each square’s spatial logic shapes accessibility and sociability patterns. The comparative analysis of movement and visual barriers using VGA metrics highlights how each urban square supports or constrains social behaviours through visibility (Fig. 4). King George Square in Brisbane reveals the highest visual integration (164.08) and visual connectivity (4515), indicating the most visually permeable environment. In contrast, Madrid’s Plaza Mayor, despite its high movement integration, shows a lower visual clustering coefficient (0.90) and visual control (1.11), potentially impeding reciprocal visual engagement. Interestingly, Isfahan’s Naghshe Jahan Square, while having low visual integration and connectivity, presents a relatively high visual clustering coefficient (0.98) and visual control (1.55), suggesting a compact visual field that promotes localised interactions over expansive visual access. Ultimately, visibility should be considered a distinct but complementary factor to movement when assessing spatial support for sociability.

#### Empirical observations coupled with computational simulations

**Fig. 5:**
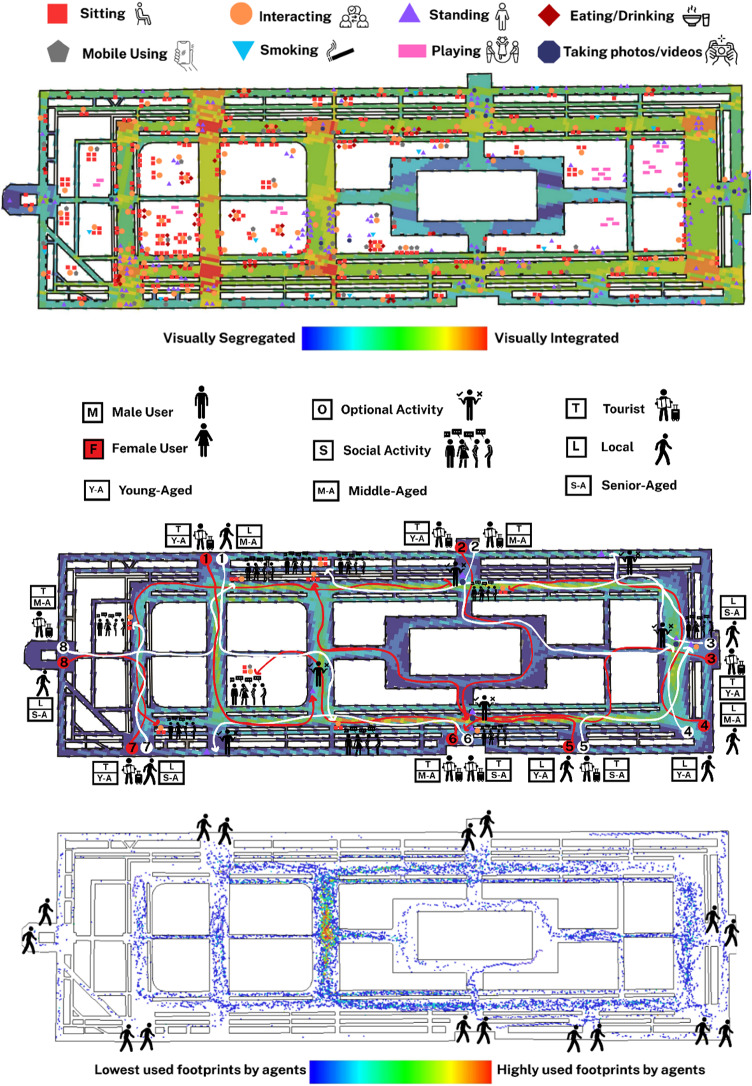
(**A**: Top): Behavioural map obtained from the observation of static activities in Naghshe Jahan Square; (**B**: Middle): Behavioural map of people-tracing in Naghshe Jahan Square, Isfahan; (**C**: Bottom): Agent-based simulation of pedestrian movement in Naghshe Jahan Square (Developed by corresponding author and M.D.).

The results obtained from behavioural observations within the Iranian case, Naghshe Jahan Square in Isfahan indicated that the spatial distribution of static behaviours reveals clear patterns in relation to the square’s spatial configuration and visual integration values (Fig. 5A). Behaviours such as sitting, interacting, standing, and taking photos are most densely concentrated in zones with moderate to high visual integration (represented in warmer colours on the VGA background), particularly around the lateral axis and near major entrances and landmarks. Areas with high visibility tend to attract more varied and denser behaviour types, especially sitting and interacting, which are heavily clustered along the garden edges around the square. The corners and visually segregated parts of the square show fewer behavioural markers, highlighting the influence of spatial accessibility on sociability. This correlation between behaviour and VGA suggests that users gravitate toward spaces where visibility supports orientation, comfort, and opportunities for social interaction. Furthermore, areas adjacent to key visual trajectories, particularly those leading toward the main architectural landmarks, appear to function as social attractors, where standing and photography activities are highly concentrated.

The people-tracing behavioural map of Naghshe Jahan Square reveals significant relationships between movement trajectories, built environment features, and resulting behaviours, categorised as either optional or social (Fig. 5B). The traced routes of 16 participants (8 male and 8 female users across varied demographic groups) demonstrate that visually integrated zones and functional attractors, such as benches, shops, landmarks, or horse-drawn carriage stations, serve as behavioural anchors that guide pedestrian flow toward specific activities. Optional behaviours, such as standing or pausing for transport or viewing store displays, tend to occur at nodes where physical affordances or spatial narrowing encourage temporary stops. In contrast, social behaviours, including sitting and interacting, are more often associated with shaded seating areas, garden edges, and visually permeable spots where sightlines encourage lingering and mutual visibility. Notably, female users (red arrows) display a stronger tendency toward areas with enhanced environmental comfort and spatial enclosure, often engaging in social interaction, while male users (white arrows) show more linear trajectories oriented toward purposeful optional stops. The conducted observations elucidated that senior locals favour accessible, and shaded spots for resting, whereas younger tourists tend toward optional activities near experiential landmarks.

The agent-based simulation map of Naghshe Jahan Square highly aligns with empirical people-tracing observations, confirming syntactical models’ predictive validity for movement behaviours (Fig. 5C). The densest agent footprints (yellow to red) closely match real pedestrian routes, especially around central areas and key intersections near entrances and visual attractors. These zones also saw convergence in people-tracing observations, suggesting configurational attributes like visual permeability and spatial accessibility heavily influence both simulated and actual movement. The overlap validates the computational simulation as a reliable analytical tool. Peripheral paths and interior fountain pool areas with minimal agent footprints mirrored low actual user traffic, highlighting spatial barriers.

**Fig. 6:**
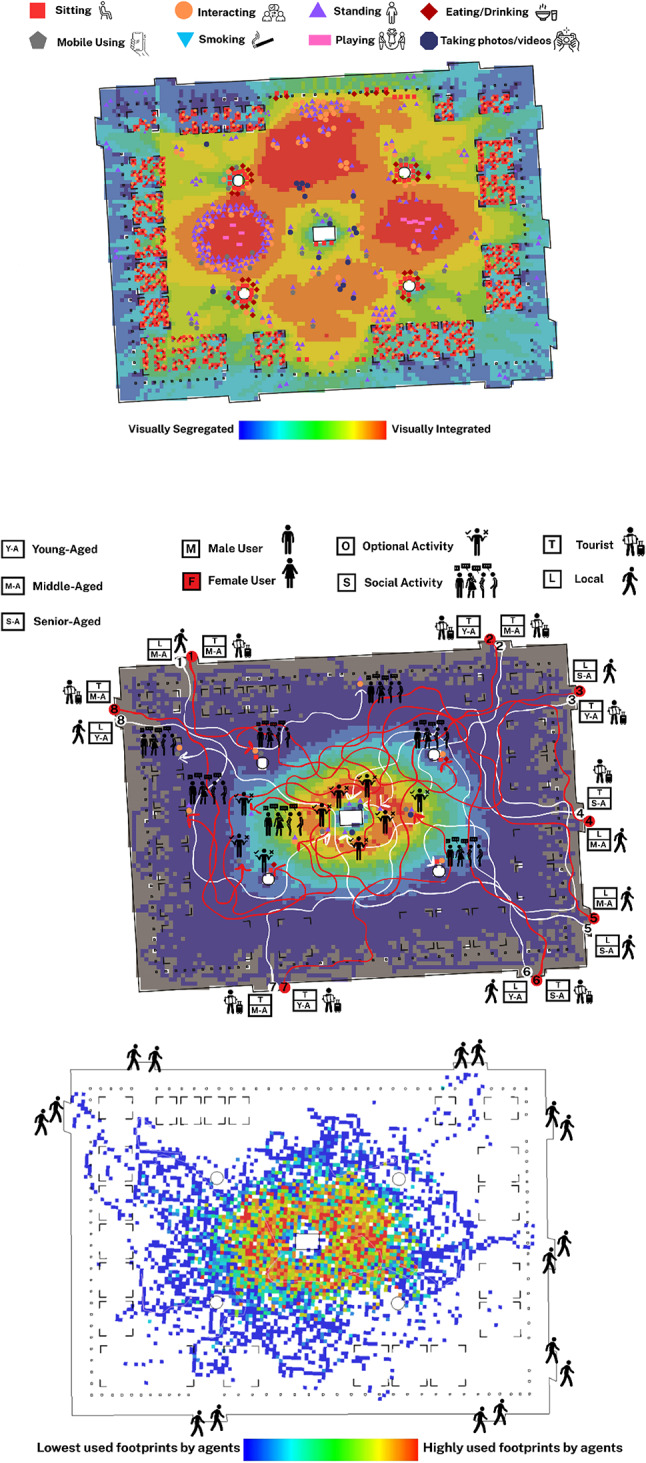
(**A**: Top): Behavioural map obtained from the observation of static activities in Plaza Mayor, Madrid; (**B**: Middle): Behavioural map of people-tracing in Plaza Mayor, Madrid; (**C**: Bottom): Agent-based simulation of pedestrian movement in Plaza Mayor, Madrid (Developed by corresponding author and M.D.).

The behavioural map of Plaza Mayor in Madrid reveals a dynamic spatial distribution of static behaviours, with significant activity concentrated along the shaded perimeter arcades and near the café umbrellas that line the edges of the square (Fig. 6A). Sitting behaviours, represented by red squares, are densely clustered around these semi-open cafés, reflecting the role of commercial amenities in encouraging longer stays. Standing (purple triangles), interacting (orange circles), and mobile phone usage (gray pentagons) are also most frequent along the outer edges and corners of the square, where foot traffic is concentrated. In contrast, the inner zones, particularly around the equestrian statue, exhibit a mix of standing, photo-taking (navy octagons), and interacting behaviours, suggesting the area’s strong visual pull and symbolic centrality. The VGA background reinforces these findings, with high visual integration (warm colours) correlating with areas of frequent social behaviours. The visually segregated corners (cooler tones) host fewer behavioural events, affirming the connection between spatial visibility and behavioural density.

The people-tracing behavioural map of Plaza Mayor in Madrid reveals how built environment features distinctly guide pedestrian trajectories toward either optional or social activities, with visible differentiation across demographic profiles (Fig. 6B). The central equestrian statue acts as a strong visual attractor for many users, especially tourists, most notably among female, middle-aged users, who tend to wander through the square before engaging in passive, optional behaviours such as standing and watching. In contrast, local users, particularly middle-aged males, are more inclined to directly approach socially vibrant zones, where they engage in social interactions, often in proximity to shaded seating areas or clustered groups, suggesting a greater familiarity with the square’s social landscape. The shaded café areas positioned along the periphery emerge as important anchors for both movement convergence and social behaviour, with multiple users’ routes terminating near these semi-private zones. The overall pattern illustrates that women more often indicate exploratory movement trajectories, while men tend to approach specific social nodes more directly. The VGA background supports these interpretations, as many of the routes align with areas of higher visual integration, confirming that movement patterns and behavioural destinations are strongly influenced by the visual and spatial characteristics of the environment.

The agent-based simulation of Plaza Mayor highly aligns with empirical people-tracing, confirming strong consistency between simulated and actual pedestrian behaviour (Fig. 6C). The central zone around the equestrian statue was the most traversed by agents, mirroring real-world users drawn to this landmark. Footprint intensity decreased towards the square’s corners and peripheral entrances, correlating with limited behavioural endpoints observed. The simulation confirms that spatial characteristics, especially high visual integration and symbolic focal points, critically shape natural movement in Plaza Mayor.

**Fig. 7:**
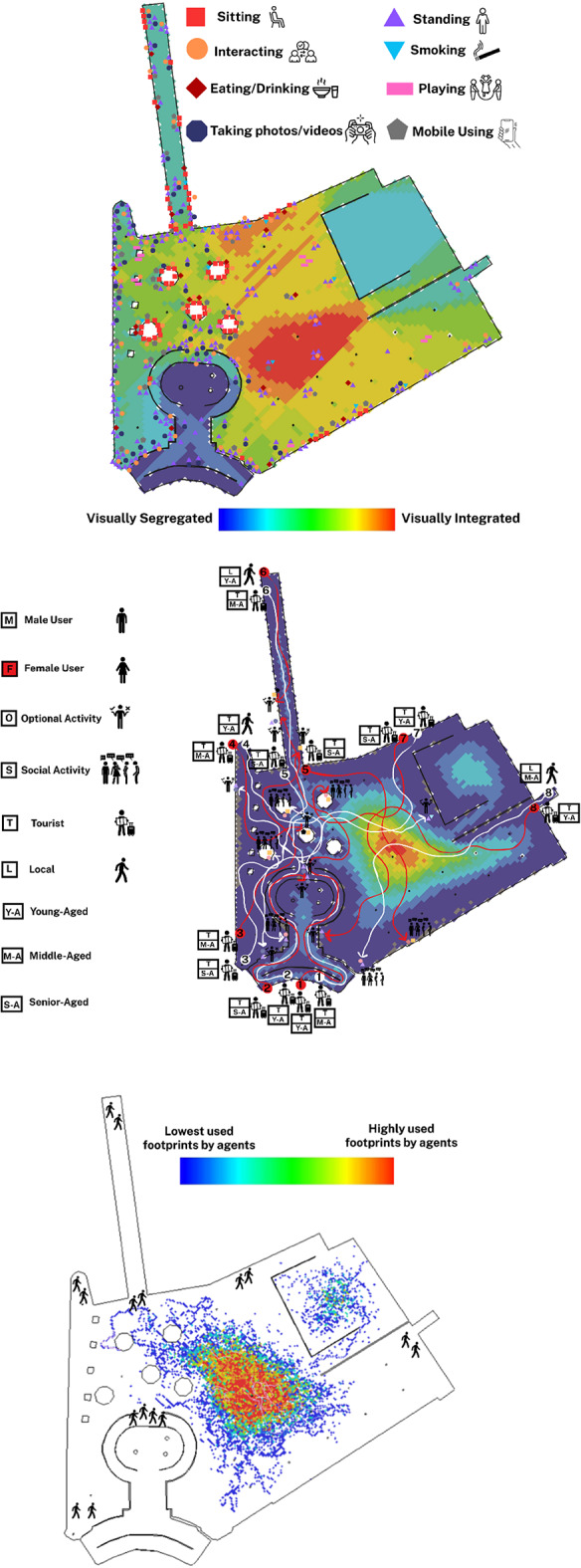
(**A**: Top): Behavioural map obtained from the observation of static activities in Bastione di Saint Remy, Cagliari; (**B**: Middle): Behavioural map of people-tracing in Bastione di Saint Remy, Cagliari; (**C**: Bottom): Agent-based simulation of pedestrian movement in Bastione di Saint Remy, Cagliari (Developed by corresponding author and C.G.).

The behavioural map of Bastione di Saint Remy in Cagliari reveals a somewhat dispersed yet concentrated pattern of static behaviours, particularly around visually integrated zones highlighted by the VGA background (Fig. 7A). Most sitting behaviours are clustered along the perimeter, particularly near the outdoor seating arrangements, with a notable density in the edges of the square. Interacting, standing, and mobile use behaviours also appear aligned with seating clusters, although the central open plaza, despite high visual integration, shows relatively low activity. This suggests a gap between spatial characteristics and actual user engagement. Unlike previous cases, social activities here appear fragmented, with less clear correspondence with respect to a behavioural epicentre dominating the square. The presence of children playing is modest and dispersed, mostly in semi-integrated zones away from main sightlines, indicating possible visual privacy or isolation preferences.

The people-tracing map of Bastione di Saint Remy in Cagliari illustrates how the built environment and demographic variables converge to shape pedestrian behaviour (Fig. 7B). While visually integrated zones, especially toward the central zone, offered higher spatial visibility, many users, particularly tourists, were drawn more strongly to panoramic viewpoints than to spatially integrated nodes. Several routes culminated at the square’s edge, where the lookout terrace provides expansive views, enhancing optional behaviours such as sitting and gazing. This tendency was particularly evident among young and middle-aged female tourists, who sought solitude and aesthetic experience over interaction. In contrast, male users, especially middle-aged tourists, were more likely to pause at seating areas or in collective zones, transitioning toward social behaviours such as conversation or resting in groups. Although the main movement corridors did not entirely align with VGA predictions, behavioural destinations tended to cluster around experiential or functionally supportive elements, such as accessible seating, edge conditions, and vistas. These findings suggest that while spatial configuration sets a foundation for movement, sensory and experiential stimuli significantly mediate how different demographic groups engage in optional versus social activities within culturally and visually rich spaces.

The agent-based simulation for Bastione di Saint Remy in Cagliari shows movement concentrated in visually integrated central areas (Fig. 7C). However, a key difference from real-life people-tracing emerges: while agents follow spatial integration, actual pedestrians gravitate towards experiential and scenic zones like the panoramic terrace, even if they’re visually less integrated. This inconsistency suggests the current design does not fully capitalise on the square’s configurational potential, as human behaviour introduces variability driven by personal goals, sensory preferences, and social intentions. Agent footprints show potential movement, while actual behaviours reflect the lived urban experience shaped by both space and content.

**Fig. 8:**
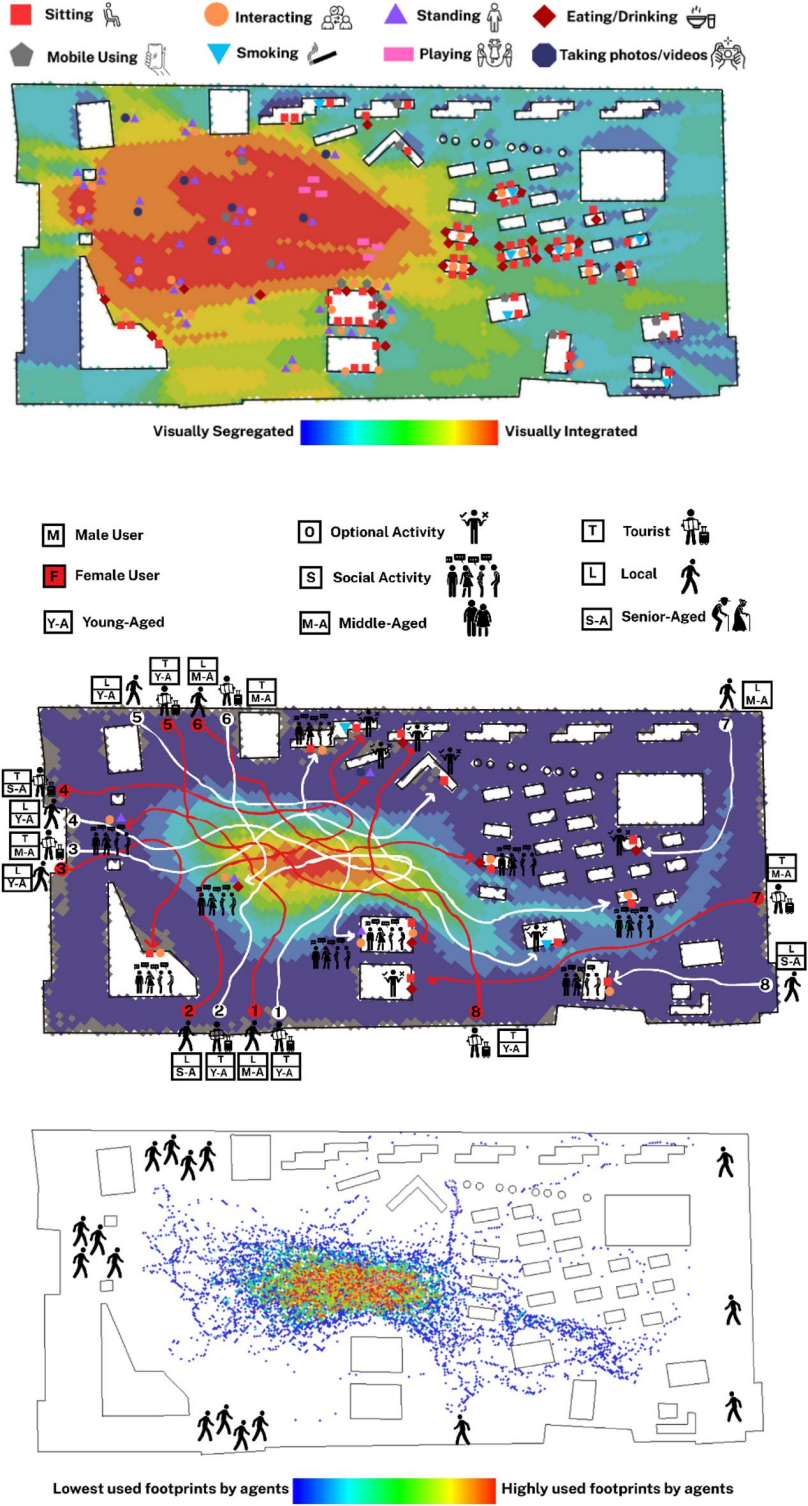
(**A**: Top): Behavioural map obtained from the observation of static activities in King George Square, Brisbane; (**B**: Middle): Behavioural map of people-tracing in King George Square, Brisbane; (**C**: Bottom): Agent-based simulation of pedestrian movement in King George Square, Brisbane (Developed by corresponding author and E.M.K.).

The behavioural map of King George Square in Brisbane reveals a clearly zoned spatial organisation of static behaviours, strongly shaped by the square’s design layout (Fig. 8A). Most sitting, eating, and interactive behaviours are clustered in the right-hand portion of the square where semi-open cafés and structured seating areas are located. These areas correspond closely with mid-to-low VGA integration zones, indicating relative visual accessibility. Conversely, the central open space, characterised by its visual dominance and spatial openness, is less densely populated by static behaviours but serves as a dynamic field for movement and flexible event-based uses. The structured and deliberate zoning allows for a spatial balance between high-flow pedestrian movement and localised zones for rest and engagement. Behaviourally, the visibility and permeability of the square guide users toward these predefined zones, producing a functionally coherent behavioural pattern across the space.

The people-tracing map of King George Square in Brisbane reveals a relatively well-structured relationship between spatial configuration and the emergence of both optional and social activities (Fig. 8B). Most pedestrian paths, regardless of gender or age group, converge within visually integrated zones, particularly the central spine and areas in proximity to the landmark City Hall. These spaces tend to host social gatherings, informal interactions, and collective sitting behaviours. For example, young male tourists (e.g., trajectory 1 white) gravitated toward communal seating for group interactions, while middle-aged local women (e.g., trajectory 1 red) were drawn toward landmark-oriented zones for social standing activities. These behaviours suggest that spatial visibility and openness, paired with landmark attraction, enhance the likelihood of social activity formation. Meanwhile, some users, particularly senior-aged or middle-aged tourists, sought peripheral benches for individual rest or small-group conversations, reflecting a pattern of optional behaviours linked to semi-enclosed or shaded environments. The gender balance of traced individuals shows consistent movement patterns between men and women, although women more often paused at landmark points, likely due to perceived safety or comfort in high-visibility, centrally located zones.

The agent-based simulation for King George Square in Brisbane strongly aligns with observed people-tracing movement patterns (Fig. 8C). The highest concentration of simulated agent footprints is on the central axis, mirroring where most real-life users converged. This central zone acts as a highly integrated spatial configuration, offering clear lines of sight and direct connectivity to major destinations like City Hall. While agents followed routes of least resistance, real users occasionally deviated due to environmental cues like shaded spots or cafés. Nonetheless, the overall overlap confirms that the square’s syntactical properties, especially visual integration and permeability, fundamentally shape pedestrian circulation, with minor deviations driven by context-specific preferences.

**Fig. 9:**
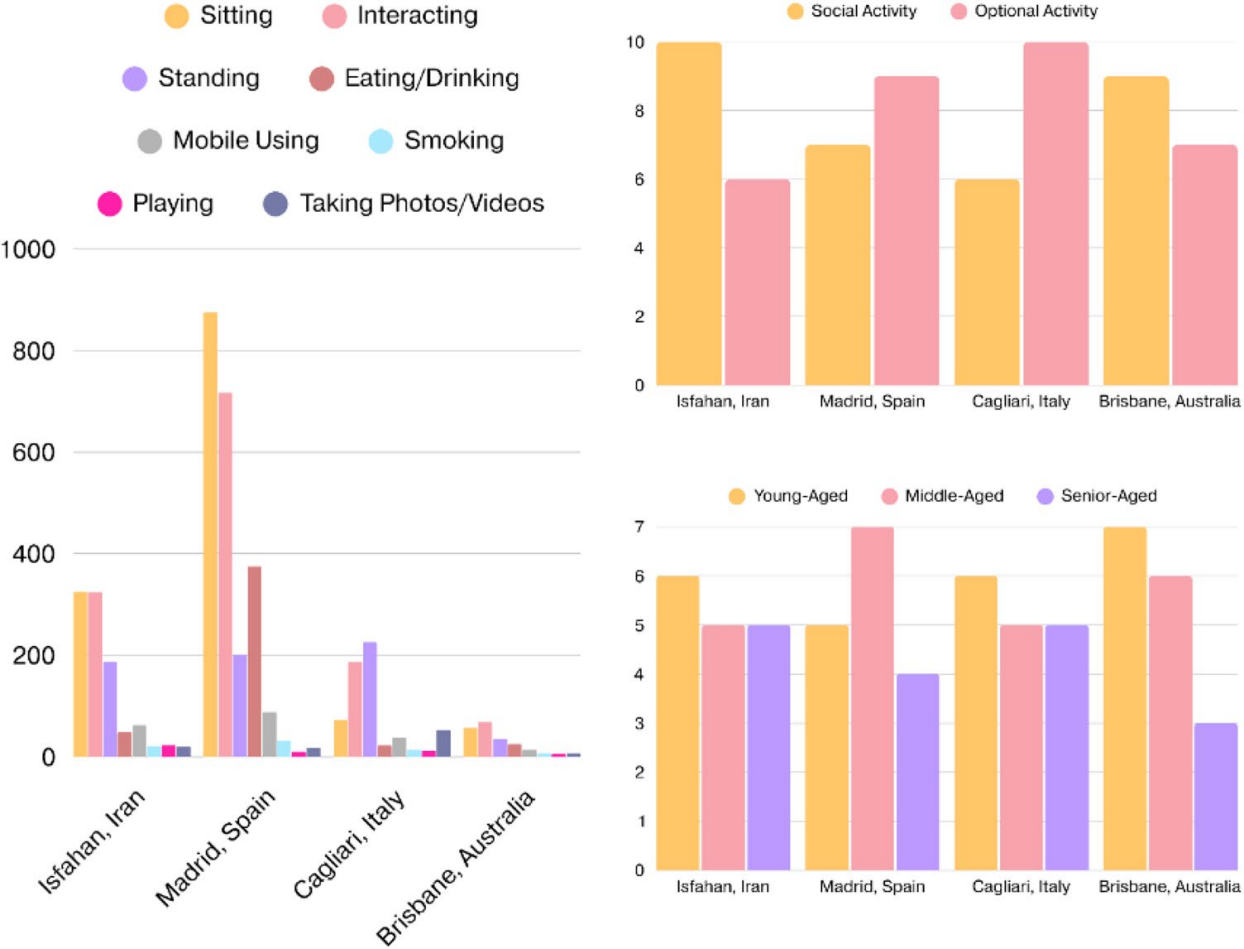
(**A**: Left): The frequency of static social behaviours across the four studied urban squares; (**B**: Top-right): The frequency of social versus optional activities across the four studied urban squares; (**C**: Bottom-right): The frequency of age group distribution across the four studied urban squares (Developed by corresponding author).

The bar chart depicting the frequency of static social behaviours across the four studied urban squares, Isfahan (Iran), Madrid (Spain), Cagliari (Italy), and Brisbane (Australia), reveals notable variation in activity intensity and distribution (Fig. 9A). Plaza Mayor in Madrid demonstrates the highest overall behavioural density, particularly in sitting, interacting, and eating/drinking behaviours, clearly indicating the square’s function as a highly sociable and economically vibrant space. This can be directly linked to its spatial openness and presence of semi-open cafés, which invite longer-term stays. Conversely, Brisbane and Cagliari display comparatively modest levels of social behaviours, with Brisbane showing minimal activity in all categories despite its organised layout. Isfahan’s square shows a relatively balanced distribution, especially in sitting and interacting, suggesting a culturally rooted use of public space even with less formal infrastructure.

The bar charts present a comparative overview of the frequency of social versus optional activities, as well as the age group distribution across the four case studies. The first chart reveals that the Iranian case had the highest incidence of social activities, suggesting an organised spatial layout, coupled with sufficient spatial facilities. In contrast, the Italian case displayed the highest rate of optional activities, highlighting a more individualistic or purpose-driven engagement with the space. Madrid and Brisbane indicated relatively balanced patterns, though Madrid leaned slightly toward optional activities and Brisbane toward social ones, reflecting how café culture and visual openness respectively shaped their spatial use (Fig. 9B). The data obtained from age group distribution shows that across all study areas, young-aged and middle-aged users dominate public space usage. Meanwhile, senior-aged participation is relatively uniform, except in Brisbane where it drops significantly, potentially reflecting discomfort or safety concerns (Fig. 9C).

## Discussion

The data obtained from this study offers a wide range of striking outcomes, yielding notable interpretative insights applicable in urban design for managing and controlling social behaviours, especially through a cross-cultural lens. Primarily, the spatial analysis elaborated on the significant role of enclosure or openness of urban squares, which could substantially influence their function as the main social hub of the broader urban fabric. This suggests that despite the potential of architecturally enclosed urban squares (as seen in Isfahan and Madrid) for creating historically significant spaces, their enclosed nature may limit their ability to become primary accessible trajectories for movement and broad social interaction within the larger urban system. They might serve more localised or destination-based functions rather than acting as highly permeable and accessible social centres for the entire neighbourhood.

The VGA outcomes interpret how an urban square’s architectural prototype dictates its visual accessibility and the resulting sociability. Plaza Mayor in Madrid, with its high visual integration and connectivity, exemplifies an environment designed for broad public awareness and spontaneous encounters, where the vast open space fosters widespread visibility. In contrast, the Iranian square, despite lower integration and connectivity, achieves high visual control and clustering, suggesting a deliberate spatial strategy to segment the visual field and encourage more focused, potentially intimate, social interactions within its private garden sections. This is a crucial finding: The Persian garden prototype, with its private segments, actively supports more localised and perhaps even private social gatherings in contrast to the more open Western typologies. This is not a deficit in sociability, but rather a different kind of sociability, one characterised by focused encounters rather than broad public engagement.

Beyond general behavioural patterns observed in the Iranian Square, the map reveals culturally specific behaviours that reflect the unique socio-spatial practices of the Iranian context. Notably, despite the deliberate urban design that delineates walkways and green zones through edge treatments and paving materials, users frequently traverse and occupy garden areas for leisure, playing and picnicking. This behaviour is further emphasised by the clustering of sitting, smoking shisha, standing near souvenir shops or the horse-drawn carriage station and playing activities along the soft edges of the gardens, which function as both physical supports and social perches. The tracing data further distinguishes between genders, with female users favoring more comfortable and enclosed spaces for social interaction, while male users demonstrate more direct, purposeful trajectories toward optional stops.

Moving to Plaza Mayor, its high overall behavioural density, especially for sitting and interacting, clearly illustrates the power of commercial amenities and semi-open cafés in cultivating prolonged stays and vibrant social life. The presence of street performers further amplifies its role as a dynamic social magnet, creating ephemeral nodes for impromptu gatherings. Yet, the critical finding of limited permanent public seating reveals a significant design shortfall; this forces users to remain standing, potentially compromising comfort and inclusivity, especially for vulnerable populations. The tracing data here further highlights how local users, particularly middle-aged males, elucidate a greater familiarity, directly heading to social zones, while female tourists often undertake more exploratory movements before engaging in passive observations, underscores how familiarity and perceived safety influence movement patterns.

The Bastione di Saint Remy offers an intriguing case where strong visual attributes like panoramic viewpoints primarily attract optional activities such as gazing and photo-taking, especially among young and middle-aged female tourists seeking solitude. Despite high visual integration in some central areas, the lack of shaded seating and functional diversity appears to undermine its potential for sustained social engagement, leading to fragmented activity patterns. This suggests that breathtaking views alone are insufficient to bolster robust social activity; they must be complemented by supportive urban furniture and diverse programming. The data further emphasises how sensory and experiential stimuli can mediate engagement, with users drawn to vistas over purely spatially integrated nodes.

Finally, King George Square presents a clear tension between intended design and socio-economic realities. While its structured zoning and high visibility generally facilitate a coherent pattern of movement and targeted social activities around landmarks like City Hall, the significant presence of homeless individuals on communal seating platforms inadvertently transforms intended social spaces into zones of avoidance for other users. This critically underscores that urban design cannot exist in a vacuum; social inequities and their spatial manifestations directly impact the usability and perceived safety of public spaces, potentially deterring broader public engagement^52^. Despite this, its openness and landmark attraction effectively draw diverse users, and the tracing data consistently shows convergence within visually integrated zones for social gatherings, indicating the foundational role of visibility and openness in promoting sociability.

In summary, the results reveal that accessible urban squares do not necessarily lead to sociable urban squares; rather, the activation of urban public squares for sustained social life is a complex interplay of cultural practices, the strategic provision of functional amenities, and the imperative to address socio-economic challenges that manifest within these shared environments. In essence, while spatial configuration and visual accessibility lay the groundwork for human behaviour, the obtained data corroborate that successful public spaces are those that holistically integrate design, culture, and inclusive management.

These findings highlight practical implications for urban planners and designers, such as the importance of integrating semi-open commercial spaces (e.g., cafés), ensuring sufficient public seating, incorporating shaded areas, and designing flexible multi-functional spaces that respond to both cultural preferences and everyday social needs. Moreover, planners must consider socio-economic factors like homelessness and security, which can significantly shape how public spaces are perceived and used. Notably, by combining quantitative syntactic modelling with qualitative behavioural observations, this study demonstrates how ‘space’ and its physical configuration evolves into ‘place’ through the lived, culturally embedded behaviours of its users, addressing a fundamental discourse in urban studies on how design, culture, and social life converge to create meaning.

Although previous studies have thoroughly investigated the relationship between spatial configuration and social behaviours in urban spaces, most have remained geographically localised and context-specific, focusing on isolated case studies within a single cultural or geographical setting. Scholars such as Can & Heath^53^, Safari & Moridani^54^, Bendjedidi et al.^55^, Mohamed et al.^56^ and Li et al.^57^ emphasised the influence of spatial integration, connectivity, and visibility on social interaction patterns. Others, including Zerouati & Bellal^58^, Sheng et al.^59^, Mohamed et al.^60^, Tahroodi & Ujang^61^ and Yu et al.^62^ explored how spatial accessibility, visual permeability or environmental features stimulate community socio-behavioural engagement. These contributions have significantly advanced the theoretical linkage between built environment and social function, yet they primarily remain confined to singular urban morphologies, often without addressing the nuances of contextual variability or user diversity.

This study expands the frontier of current knowledge by adopting a comparative cross-cultural lens, integrating spatial syntax analysis with empirical behavioural observations across four distinct cultural contexts: Iran, Spain, Italy, and Australia. Unlike prior research, which has largely remained bounded to single-site analyses, this study systematically examines how different spatial prototypes, such as organic, semi-organic, and grid-based configurations, interact with culturally ingrained behavioural norms and socio-demographic dynamics. It bridges syntactical modelling with lived human behaviours by incorporating dynamic people-tracing and agent-based simulation, revealing not only how people move through space, but also why and under what contextual or social stimuli. The originality of this research lies in its ability to contextualise spatial metrics within broader socio-cultural frameworks, offering a multi-scalar, multi-method, and multi-cultural perspective that has been largely absent in the field. In doing so, this research contributes a more globally nuanced and behaviourally grounded understanding of how spatial design can promote inclusive, culturally responsive, and socially vibrant public spaces.

Despite its contributions, this study has certain limitations. The observations were conducted under fair weather and during specific time frames, which may not fully capture seasonal or temporal variations in behaviour. Additionally, while four culturally diverse contexts were examined, the findings may not be universally applicable to all urban environments, particularly in American, African, or East Asian contexts. Future research should explore a wider range of cities, integrate longitudinal observations, and incorporate additional factors such as climatic conditions, socio-economic inequalities, and technological interventions to better understand the evolving dynamics of public spaces.

### Practical implications for policy and planning

This study provides actionable insights for urban planners, designers, and policymakers striving to create socially vibrant and culturally inclusive public spaces. A key implication is that spatial accessibility alone does not guarantee sociability; design interventions must address the interaction between spatial configuration, cultural practices, and functional amenities. For example, the success of Plaza Mayor highlights how semi-open commercial spaces and diversified seating arrangements extend dwell time and encourage interaction, while the limited functional diversity in Bastione di Saint Remy demonstrates the risks of overlooking amenities despite high visual potential.

Urban planners should prioritise visual permeability and flexible multi-functional zones, enabling both spontaneous encounters and private gatherings. In culturally conservative contexts such as Iran, localised visibility and shaded seating enhance comfort for women and families, emphasising that context-sensitive visibility strategies outperform uniform design templates. Policymakers can integrate these lessons into urban revitalisation programs by combining physical upgrades with cultural programming—for instance, events or performances that animate open areas without excluding quieter uses.

Local governments must also consider socio-economic realities, such as homelessness or informal uses, which were found to alter perceived safety and usability, particularly in Brisbane. Addressing these challenges requires coordinated urban governance, aligning public space design with social services and management strategies. Overall, this cross-cultural evidence underscores the need for holistic, user-focused, and culturally adaptive planning, enabling public spaces to become inclusive arenas for community life rather than mere physical nodes of circulation. Building on these practical insights, the following conclusion summarises the key contributions and outlines the study’s limitations and future research opportunities.

## Conclusion

This study aimed to examine the socio-behavioural variations of pedestrians in urban public squares across four distinct cultural contexts by identifying the interaction between spatial configuration and socio-behavioural patterns. By employing a multi-method approach involving space syntax analysis, empirical observations like behavioural mapping and people-tracing, coupled with agent-based simulations, this research provides a multifaceted understanding of how spatial and visual attributes influence public life. The findings elucidate that the accessibility of urban squares is not necessarily determinative of their social vibrancy. Rather, fostering sustained social life in these environments is governed by an intricate nexus of factors, including culturally specific behaviours, the purposeful allocation of functional provisions, and the critical amelioration of socio-economic challenges inherent to their shared public domain.

While spatial configuration lays the structural foundation for movement and visibility, the actual use of public squares is mediated by cultural preferences, social practices, and functional amenities. For instance, enclosed squares in places like Isfahan and Madrid tend to cultivate more localised and culturally sensitive social interactions, whereas open-configured squares, such as Brisbane’s, support broader movement and visual connectivity. Importantly, visual integration and clustering coefficients emerged as strong predictors of dynamic and static socio-behavioural density respectively, while social and optional activities were shaped by a combination of visual access, environmental comfort, and cultural familiarity. These insights not only underline the importance of designing inclusive and culturally sensitive public spaces that balance spatial logic with social and functional diversity, but also offer key takeaways for developing urban public spaces that truly resonate with local cultures and promote lively social behaviours within urban public squares.

The originality of this research lies in its cross-cultural comparative approach, bridging spatial analysis and lived human behaviours across four diverse urban contexts, an effort that largely absent in previous studies. Whereas earlier works have typically focused on isolated case studies and generalised spatial theories, this study contextualises spatial syntax within socio-cultural realities, reflecting how cultural customs, demographic variables, and urban governance influence spatial usage. This cross-contextual application emphasises the necessity of adapting spatial design to the cultural and behavioural needs of local populations. By contextualising space syntax within cultural and behavioural frameworks, this study highlights how the transformation of ‘space’ into ‘place’ is shaped by socio-cultural practices, governance, and urban design strategies. Ultimately, this study accentuates that accessible design alone does not guarantee sociability. Successful public squares emerge from an integrative approach, one that synthesises spatial logic with cultural sensitivity, inclusive programming, and equitable governance to promote resilient, engaging, and human-centred public spaces.

Despite offering significant contributions to the understanding of the socio-behavioural variations of public squares in different cultural and spatial settings, it is important to acknowledge certain limitations. Although the selection enriches cross-cultural comparisons, the findings may not fully represent the cultural domain of the studied countries, or capture the broader spectrum of urban squares in other geographic regions or under differing governance and socio-political structures. While acknowledging the time-consuming and expense-driving nature of adopting such cross-cultural studies, the current results could be expanded or contrasted with American, African, or East Asian contexts to provide a more generalisable understanding of these cross-cultural socio-behavioural dynamics. Consequently, generalisability remains context-dependent, and caution is advised in applying the conclusions universally.

Second, the observational approach, while robust in documenting real-life behaviour, is inherently time- and context-sensitive. Variability in the time of day, weather conditions, seasonal cycles, and occasional events may have influenced behavioural outcomes, potentially limiting the consistency of comparisons across sites. Moreover, gendered behaviours were interpreted through observational proxies, which may not account for the full complexity of user motivations, internal perceptions, or cultural pressures, particularly in more socially conservative contexts like Iran. Since the users of the studied urban squares consist of both tourists and locals, the findings may not have fully captured the cultural particularities of the study area. However, they also highlight the context-based characteristics of users to portray their socio-behavioural dynamics. Furthermore, the presence of homeless individuals and informal users also introduced variables that were beyond the control or direct scope of this study but nonetheless influenced space utilisation patterns.

Additionally, while space syntax and agent-based tools offer a strong analytical basis for understanding socio-spatial logic and visual permeability, they primarily simulate theoretical movement and sightlines rather than fully capturing subjective spatial experiences, such as perceived safety, social norms, or aesthetic appeal. Agent-based simulations, despite their predictive power, may oversimplify human decision-making by abstracting cultural cues or micro-level influences. Future studies should consider incorporating virtual reality, participatory and qualitative methods, such as in-depth interviews, surveys, or ethnographic techniques, to complement spatial analyses with user-centred perspectives. Looking forward, comparative studies across a wider range of public spaces, such as marketplaces, transit nodes, linear streets or parks, could manifest additional layers for socio-behavioural insights.

## Data Availability

The data that support the findings of this study are available from the corresponding author upon reasonable request. Restrictions may apply to the availability of the data due to privacy and ethical considerations.
